# Clinical features and prognostic analysis of patients with *Aspergillus* isolation during acute exacerbation of chronic obstructive pulmonary disease

**DOI:** 10.1186/s12890-021-01427-4

**Published:** 2021-02-26

**Authors:** Yu Gu, Xianping Ye, Yu Wang, Kunlu Shen, Jinjin Zhong, Bilin Chen, Xin Su

**Affiliations:** 1 Department of Respiratory and Critical Care Medicine, Jinling Hospital, Nanjing Medical University, 305 Zhongshan East Road, Xuanwu District, Nanjing, 210002 China; 2 Department of Respiratory and Critical Care Medicine, Jinling Hospital, Medical School of Nanjing University, Nanjing, 210002 China; 3 Department of Respiratory and Critical Care Medicine, Jinling Hospital, The First School of Clinical Medicine, Southern Medical University, Guangzhou, 210002 China

**Keywords:** AECOPD, *Aspergillus* isolation, Pulmonary aspergillosis, Colonization, Prognosis

## Abstract

**Background:**

Lower respiratory tract (LRT) specimen culture is widely performed for the identification of *Aspergillus*. We investigated the clinical features and prognosis of patients with *Aspergillus* isolation from LRT specimens during acute exacerbation of chronic obstructive pulmonary disease (AECOPD).

**Methods:**

This is a 6-year single-center, real-world study. 75 cases out of 1131 hospitalized AECOPD patients were positive for *Aspergillus*. These patients were carefully evaluated and finally diagnosed of pulmonary aspergillosis (PA, 60 cases, 80%) or colonization (15 cases, 20%). Comparisons of clinical data were performed between these two groups. A cox regression model was used to confirm prognostic factors of *Aspergillus* infection.

**Results:**

The PA group had worse lung function and higher rates of systemic corticosteroid use and broad-spectrum antibiotic use before admission than the colonization group. The PA group had significantly higher in-hospital mortality and 180-day mortality than the colonization group (45% (27/60) vs. 0% (0/15), *p* = 0.001, and 52.5% (31/59) vs. 6.7% (1/15), *p* < 0.001*,* respectively). By multivariable analysis among *Aspergillus* infection patients, antifungal therapy (HR 0.383, 95% CI 0.163–0.899, *p* = 0.027) was associated with improved survival, whereas accumulated dose of systemic steroids > 700 mg (HR 2.452, 95% CI 1.134–5.300, *p* = 0.023) and respiratory failure at admission (HR 5.983, 95% CI 2.487–14.397, *p* < 0.001) were independently associated with increased mortality**.** Significant survival differential was observed among PA patients without antifungals and antifungals initiated before and after *Aspergillus* positive culture (*p* = 0.001).

**Conclusions:**

*Aspergillus* isolation in hospitalized AECOPD patients largely indicated PA. AECOPD patients with PA had worse prognosis than those with *Aspergillus* colonization. Empirical antifungal therapy is warranted to improve the prognosis for *Aspergillus* infection.

## Introduction

Chronic obstructive pulmonary disease (COPD) is a common, preventable and treatable chronic respiratory disease characterized by airflow limitation and persistent respiratory symptoms. Airway microorganisms, including bacteria, viruses and fungi, play an important role in the pathogenesis of acute exacerbation of COPD (AECOPD) [1]. Recently, fungal infection and colonization, especially by *Aspergillus* spp., have been increasingly reported in COPD patients.

In some studies, the rates of isolation of *Aspergillus* spp. from COPD patients reached 16.6% and 14.1% during exacerbation and follow-up, respectively [2, 3]. Isolation of *Aspergillus* from lower respiratory tract (LRT) samples may suggest temporary fungal passage, benign carriage, colonization, or infection [4, 5]. Pulmonary aspergillosis (PA) is a life-threatening opportunistic infection. Depending on the immune status of patients and *Aspergillus* virulence, PA can be classified into different types, including allergic bronchopulmonary aspergillosis (ABPA), invasive pulmonary aspergillosis (IPA) and chronic pulmonary aspergillosis (CPA) [6]. Up to 22% of COPD patients with *Aspergillus* isolation from LRT samples have IPA [7]. IPA has a very poor prognosis in the COPD population, with a mortality rate as high as 71.7% [8].

Early recognition of PA is essential to decrease mortality and achieve a good outcome [9–11]. However, it is difficult to establish a PA diagnosis in patients with AECOPD. It is rarely feasible to perform lung biopsy in these patients [12]. LRT specimens are easier to collect than bronchoalveolar lavage fluid (BALF), which is widely used to detect *Aspergillus*. Distinguishing colonization from infection in AECOPD patients is still a challenge for clinicians.

The aim of this study was to describe clinical features of infection and colonization in AECOPD patients with positive *Aspergillus* isolation from LRT, and identify potential prognostic factors of *Aspergillus* infection on day-180 survival.

## Patients and methods

### Patient recruitment

This retrospective, real-world study was conducted among hospitalized patients with AECOPD who were admitted to Jinling Hospital (a tertiary teaching hospital with approximately 2500 + beds) from January 2014 to December 2019. The study was approved by the ethics committee of Jinling Hospital. The informed consent was obtained from all participants in written form. A diagnosis of COPD was based on the Global Initiative for Chronic Obstructive Lung Disease guidelines [13]. Hospitalized AECOPD patients with at least one qualifying LRT specimen culture were screened and enrolled. Patients were excluded if a primary diagnosis of neutropenia (< 500 neutrophils/mm^3^) or hematologic malignancy was established. The medical records of these patients, including data on clinical manifestations, comorbidities, microbiology, radiology, treatment and survival within 180 days, were reviewed.

### Definitions of pulmonary aspergillosis and colonization

The diagnosis of IPA was based on the Bulpa criteria [14]. Patients with proven and probable PA were enrolled. CPA was diagnosed according to the European Society for Clinical Microbiology and Infectious Diseases (ESCMID) and European Respiratory Society (ERS) guidelines [15]. Proven IPA was confirmed by the presence of hyphae compatible with *Aspergillus* in specimens taken from a pulmonary lesion through pulmonary biopsy within the previous 3 months. Probable IPA was diagnosed requiring three following criteria: (1) the use of steroids and routine treatment failure in severe AECOPD patients; (2) one of following microbiological evidence of *Aspergillus,* such as *Aspergillus* isolation from qualified LRT specimens, a positive serum *Aspergillus* antibody test or two sequential positive serum galactomannan (GM) tests; and (3) one or more of the following radiological imaging presentations: consolidation lesions or nodules with or without cavity formation, an air crescent sign or a halo sign. The definition of CPA was as follows: (1) persistent chronic respiratory or systemic symptoms, such as cough, hemoptysis, breathlessness, or weight loss, for at least 3 months; (2) the formation or progressive enlargement of one or more pulmonary cavities with or without a fungal ball or nodules on chest imaging; and (3) microbiological evidence of *Aspergillus* spp. from LRT specimens or the presence of positive *Aspergillus*-specific IgG; and (4) *Aspergillus* hyphae on histopathology or positive *Aspergillus* isolation from lung biopsy specimens. Bronchial lung cancer, pulmonary tuberculosis and other diseases with similar symptoms were excluded when histopathological evidence was lacking. *Aspergillus* colonization was defined as the positive isolation of *Aspergillus* from LRT specimens from patients who had no other supportive evidence of PA and recovered from AECOPD without antifungal therapy [14].

### Statistical analysis

All statistical analyses were performed using SPSS statistics software (version 25). Qualitative variables are expressed as percent distributions in each category, and quantitative variables are expressed as means ± standard deviations for normally distributed variables or medians (ranges) for nonnormally distributed variables. Pearson’s chi-square test or Fisher’s exact test was used to compare qualitative variables. Quantitative variables with normal distributions were compared with Student’s t-test, while nonnormally distributed variables were compared with the Mann–Whitney U test.

Survival curves were used to analyze the prognosis of patients from admission to day 180. Survival analysis was performed with the Kaplan–Meier method with the log-rank test. A multivariable Cox analysis with forward selection was performed in *Aspergillus* infection to determine independent factors associated with day-180 mortality. Variables with a *p* value < 0.1 in univariate analysis were included into the multivariate model. Finally, only variables with *p* value < 0.05 were retained in the multivariate model. Statistical tests were two-tailed, and a P value less than 0.05 was considered statistically significant.

## Results

There were 1298 AECOPD patients admitted to our hospital from January 2014 to December 2019. One patient was excluded because of neutropenia, and 166 patients were excluded because LRT specimen culture was not performed. A total of 1131 patients had at least one qualified LRT specimen culture. Among these, *Aspergillus* was isolated from LRT specimens from 75 (6.6%) patients. Sixty (80%) out of 75 patients were finally diagnosed with proven IPA (n = 1), probable IPA (n = 55) and CPA (n = 4) and included in the PA group. One proven IPA case was confirmed by computed tomography (CT)-guided percutaneous lung biopsy. Fifteen (20%) out of 75 patients were positive for colonization.

From the 75 PA patients, 400 cultures were performed, yielding 142 positive *Aspergillus* isolates from LRT specimens. Positive LRT specimens consisted of sputum (n = 118) and bronchial aspirate (n = 24). *A. fumigatus* was the most frequently recovered species (n = 54, 72%), followed by *A. flavus* (n = 9, 12%), *A. niger* (n = 3, 4%) and unclassified species (14, 18.7%). Two species of *Aspergillus* were isolated from LRT specimens from five patients (*A. fumigatus* + *A. flavus* (n = 3) and *A. fumigatus* + *A. niger* (n = 2)).

### Comparisons of clinical characteristics, laboratory tests and radiological presentations

The clinical characteristics of patients with positive *Aspergillus* isolation are shown in Table 1. There was no significant difference between the two groups in terms of demographic data. The PA group had a higher proportion of patients with severe COPD (GOLD III–IV) than the colonization group (*p* = 0.018). The PA group also had a higher rate of admission to the intensive care unit (ICU) during hospitalization than the colonization group (*p* = 0.028). There was no significant difference in the length of hospital stay. More patients with *Aspergillus* infection required mechanical ventilation than patients with colonization (45% vs. 13.3%, *p* = 0.024). PA patients had a higher rate of systemic corticosteroid use than colonization patients (51.7% vs 20.0%, *p* = 0.028). Prior to admission, the proportion of patients who received intravenous broad-spectrum antibiotics was higher in the PA group than in the colonization group (75.0% vs 20.0%, *p* < 0.001). The most common symptoms were cough (n = 70, 93.3%) and sputum production (n = 50, 66.7%), but no difference was observed between the two groups. Fever was more common in the PA group than in the colonization group (65% vs 26.7%, *p* = 0.007). Hemoptysis was uncommon in both groups. In terms of comorbidities, no significant difference was observed.

**Table 1:** Clinical characteristics of patients positive for *Aspergillus* isolation from LRT specimens

Variables	PA group n = 60	Colonization group n = 15	P value
Age (years, median)	71.5 (31, 93)	75 (49, 89)	0.219
Sex (male), n (%)	54 (90)	14 (93.3)	1.000
GOLD III–IV, n (%)	53 (88.3)	9 (60)	**0.018**
Corticosteroids before admission, n (%)			
Inhaled	45 (75)	9 (60)	0.335
Intravenous or oral	31 (51.7)	3 (20)	**0.028**
Accumulated dose of systemic steroids > 700 mg^a^	11 (18.3)	1 (6.7)	0.439
Broad-spectrum antibiotics before admission^b^, n (%)	45 (75)	3 (20)	** < 0.001**
Hospital stay (days, median)	15.5 (1–72)	15 (7–42)	0.691
ICU admission^c^, n (%)	31 (51.7)	3 (20)	**0.028**
Mechanical ventilation, n (%)	21 (35)	2 (13.3)	0.128
Clinical symptoms, n (%)			
Fever^d^	39 (65)	4 (26.7)	**0.007**
Cough	57 (95)	13 (86.7)	0.260
Sputum	43 (71.7)	7 (46.7)	0.066
Hemoptysis	4 (6.7)	2 (13.3)	0.593
Respiratory failure	28 (46.7)	3 (20)	0.061
Comorbidities, n (%)			
Bronchiectasis	10 (16.7)	2 (13.3)	1.000
Tuberculosis before	11 (18.3)	3 (20)	1.000
Hypertension	24 (40)	8 (53.3)	0.350
Diabetes mellitus	20 (33.3)	2 (13.3)	0.205
Cardiac insufficiency	20 (33.3)	3 (20)	0.369
Renal insufficiency^e^	8 (13.3)	2 (13.3)	1.000
Solid malignant tumor	5 (8.3)	3 (20)	0.193

*ICU* intensive care unit. ^a^Dose of systemic steroids in prednisone equivalents before first *Aspergillus* isolation; ^b^three or more antibiotics in the past 3 months; ^c^ICU admission at any time during hospitalization; ^d^T > 38℃; ^e^serum creatinine > 1.5 mg/dL; significance of bold: a P value less than 0.05 was considered statistically significant

Laboratory and CT imaging data were included in Table 2. The laboratory data showed that inflammatory biomarkers including white blood cell (WBC) counts, neutrophil counts and C-reactive protein (CRP) levels significantly increased in the PA group. The level of procalcitonin (PCT) did not differ between the two groups. Patients with PA had significantly lower albumin (ALB) than patients with colonization (28.3 ± 6.2 vs. 32.3 ± 6.5, *p* = 0.029). Serum galactomannan (GM) detection was performed in 63 patients. Serum GM (cutoff 0.5) was found to be positive in 28 (53.8%) cases in the PA group and 1 (6.7%) patient in the colonization group. The patient with positive serum GM in the colonization group was finally diagnosed with colonization due to subsequent consecutive negative GM results, and the patient recovered from AECOPD without antifungal therapy. BALF GM detection was not routinely performed in this population. All BALF GM tests in 6 patients in the PA group were positive (cutoff 0.7). CT was performed in 67 (89.3%) patients. The CT findings were not significantly different between the two groups. The results demonstrated that infiltration (n = 62) was the most common radiological finding in patients with *Aspergillus* isolation from LRT specimens. Specific signs were rarely observed in AECOPD patients with PA; only 2 patients had halo signs and 3 patients had air crescent signs.

**Table 2:** Laboratory and radiological data of patients with *Aspergillus* isolation from LRT specimens

Variables	PA group n = 60	Colonization group n = 15	P value
Laboratory data^a^			
WBC count (10^9^/L)	11.3 (3.3–43.3)	6.3 (3.3–26.5)	**0.001**
Neutrophil count (10^9^/L)	9.6 (2.3–41.9)	4.1 (2.1–23.5)	**0.001**
Lymphocyte count (10^9^/L)	0.7 (0.1–4.9)	1.1 (0.4–2.3)	0.063
Hb (g/L)	117.7 ± 19.3	128.9 ± 43.5	0.345
PLT (10^9^/L)	187 (20, 522)	222 (46, 370)	0.425
ALB (g/L)	28.3 ± 6.2	32.3 ± 6.5	**0.029**
LDH (U/L)	457.5 (155—3126)	269 (168—1096)	0.056
CRP (mg/L)	64.3 (1.1–258.8)	16.2 (0—320)	**0.006**
PCT (ug/L)	0.2 (0–16)	0.1 (0–9.6)	0.195
Serum GM^b^, n (%)	28/52 (53.8)	1/11 (9.1)	**0.007**
BALF GM^c^, n (%)	6/6 (100)	/	
Radiological data, n (%)	53 (88.3)	14 (93.3)	
Consolidation	20	3	0.349
Infiltration	51	11	0.058
Nodules	11	4	0.498
Cavitation	8	0	0.189
Halo sign	2	0	1.000
Air crescent sign	3	0	1.000
Hydrothorax	22	2	0.059
Pleural thickening	30	4	0.062

*WBC* white blood cell, *Hb* hemoglobin, *PLT* platelet, *ALB* albumin, *LDH* lactate dehydrogenase, *CRP* C-reactive protein, *PCT* procalcitonin, *IL-6* interleukin-6, *GM* galactomannan, *BALF* bronchoalveolar lavage fluid; ^a^Blood specimen; ^b^the cutoff value is 0.5; ^c^the cutoff value is 0.7; significance of bold: a P value less than 0.05 was considered statistically significant

### Prognostic factors for PA patients and antifungal therapy

**Fig. 1:**
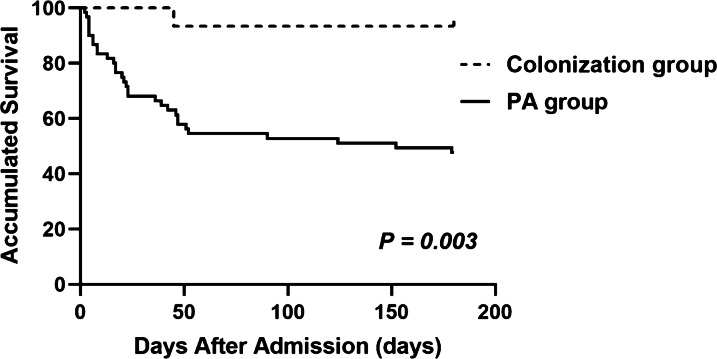
Survival from admission to day 180 in patients in the PA and colonization groups according to Kaplan–Meier analysis with the log-rank test (*p* = 0.003)

**Fig. 2:**
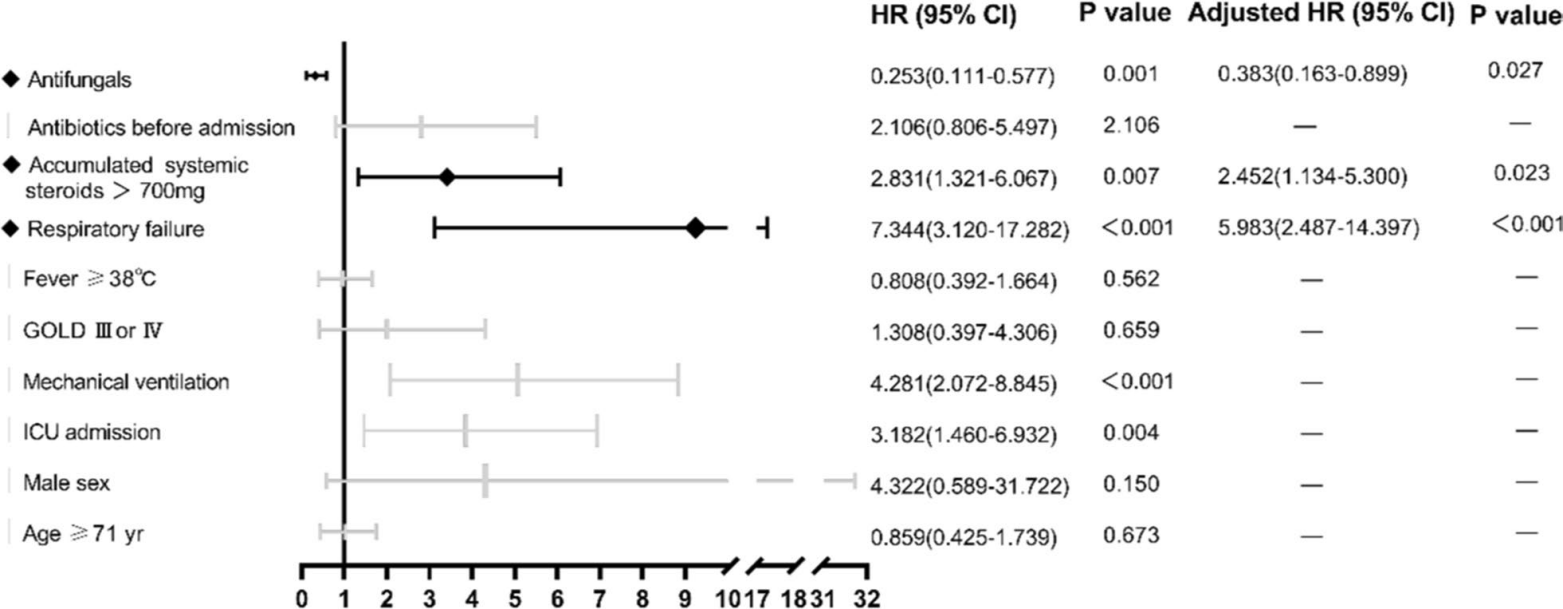
Forest plot of prognostic factors independently associated with 180-day mortality in *Aspergillus* infection patients

We followed 75 enrolled patients from admission to day 180. The clinical outcomes are presented in Table 3. The total in-hospital and 180-day mortality rates were 36% (27/75) and 43.2% (32/74), respectively, in patients with *Aspergillus* isolation from LRT specimens. Both in-hospital and 180-day mortality were significantly higher in the PA group than in the colonization group (45% vs. 0 and 52.5% vs. 6.7%, *p* = 0.001, *p* < 0.001, respectively). Among 31 patients who died in the PA group, 21 died of aspergillosis, and 10 died of aspergillosis comorbid with other diseases (such as lung cancer, renal failure, or cardiac failure). One patient in the colonization group died of multiple organ dysfunction syndrome (MODS) after meningioma surgery. The Kaplan–Meier 180-day curve revealed that patients in the PA group (105 days; 95% CI 85–125) had a shorter mean survival time than those in the colonization group (171 days; 95% CI 154–188) (log-rank, *p* = 0.003) (Fig. 1). Notably, all patients who died in the PA group had IPA. By multivariable analysis, antifungal therapy (HR 0.383, 95% CI 0.163–0.899, *p* = 0.027) was associated with improved survival, whereas accumulated dose of systemic steroids > 700 mg (HR 2.452, 95% CI 1.134–5.300, *p* = 0.023) and respiratory failure at admission (HR 5.983, 95% CI 2.487–14.397, *p* < 0.001) were independently associated with higher day-180 mortality (Fig. 2).

**Table 3:** In-hospital mortality and outcomes following 180 days in the PA group and colonization group

Outcomes	PA group n = 60	Colonization group n = 15	P value
In-hospital mortality, n (%)	27 (45)	0	0.001
Survival^a^, n	28	14	
Lost to follow-up^b^	1	0	
180-day mortality^c^, n (%)	31 (52.5)	1 (6.7)	< 0.001
Due to Aspergillosis	21	0	
Due to Aspergillosis and other causes	10	0	
Not due to Aspergillosis	0	1	

^a^Patients survived for 180 days until the end of follow-up. ^b^These patients could not be contacted because of incorrect or lost information. ^c^Death within 180 days after admission

**Fig. 3:**
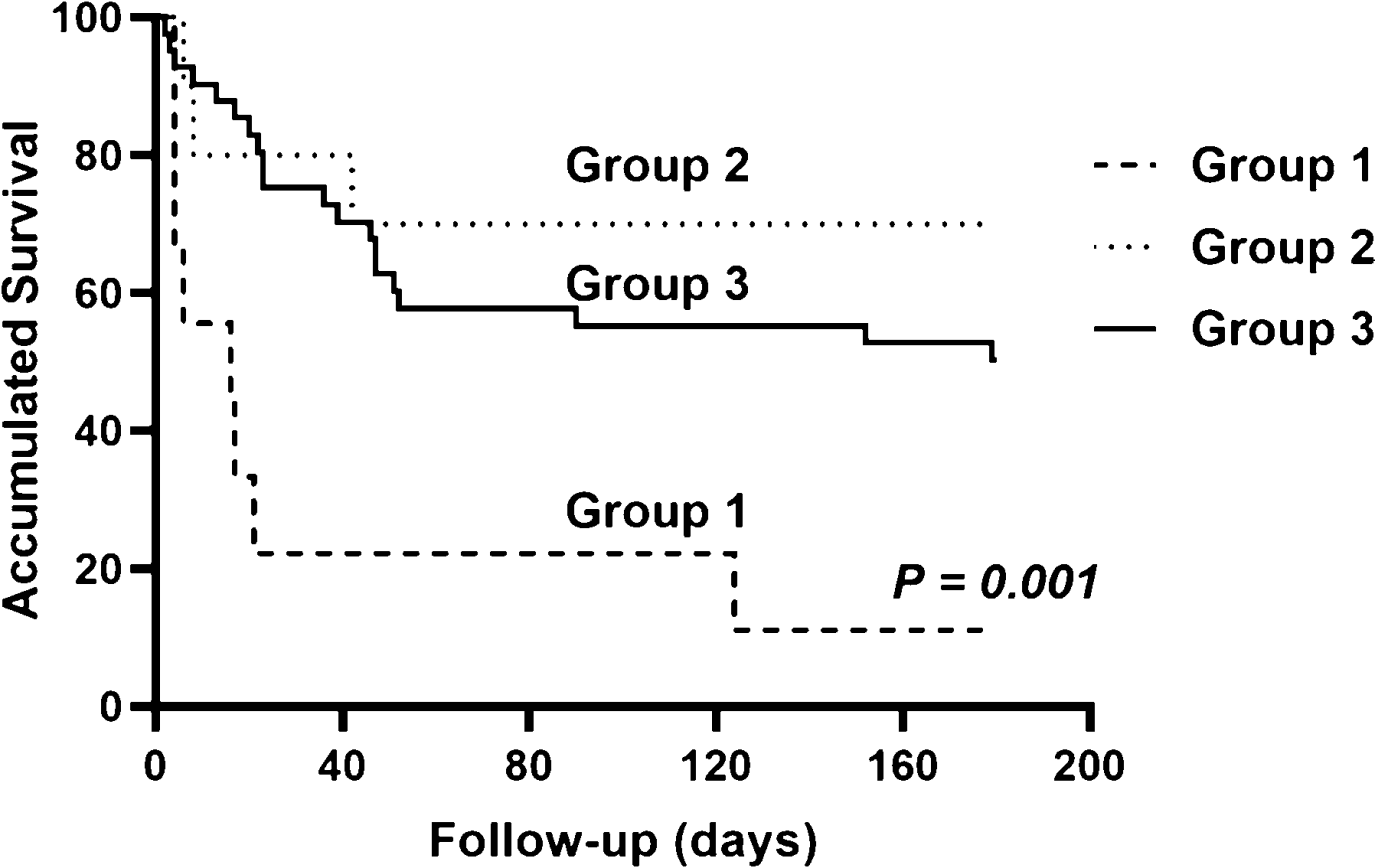
Kaplan–Meier cumulative survival curve of PA patients in the 3 subgroups. Curves were compared with the log‑rank test (*p* = 0.001)

**Fig. 4:**
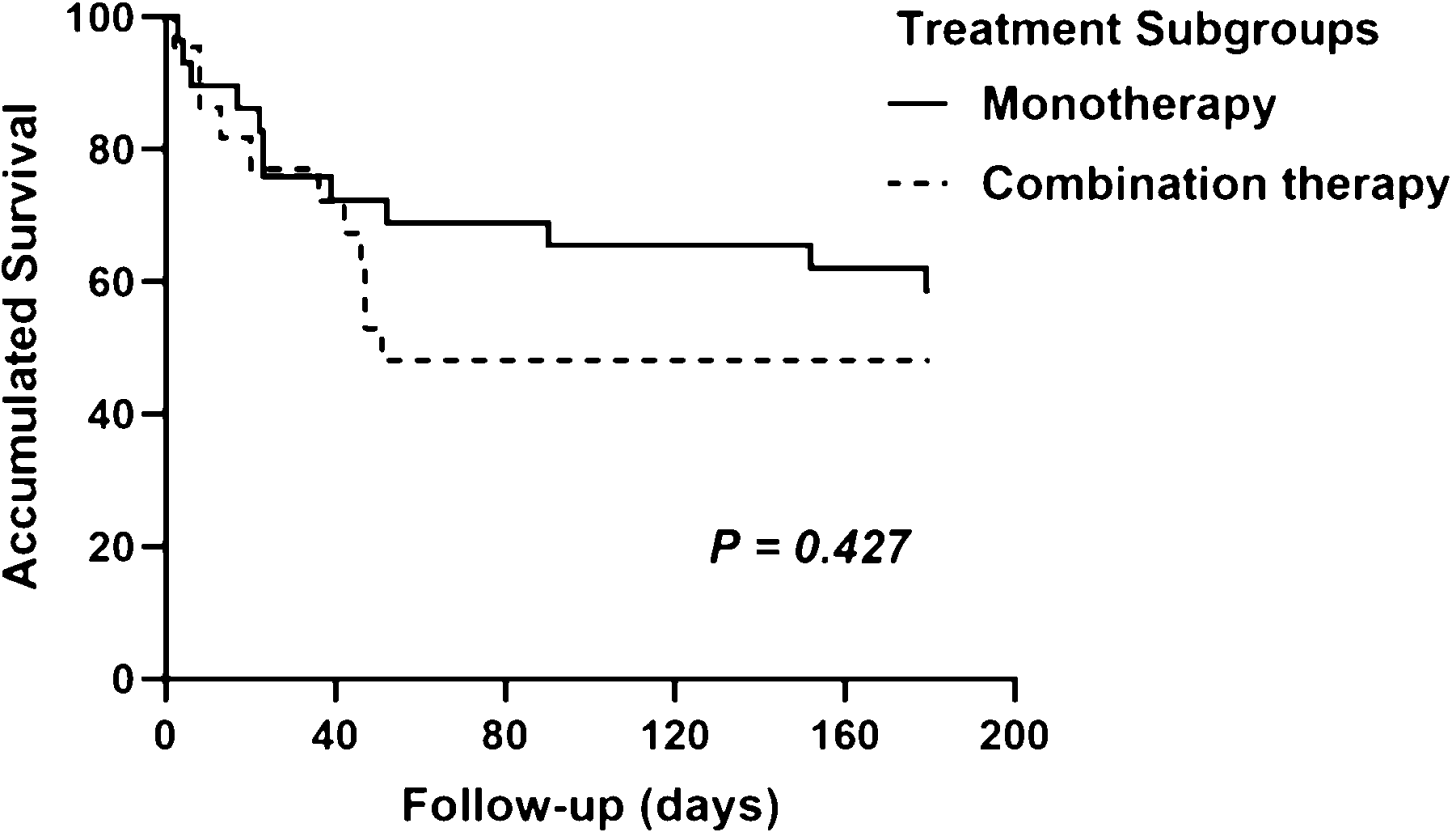
Survival from admission to 180 day of patients with monotherapy and combination antifungal therapy by Kaplan–Meier analysis with log-rank test (*p* = 0.427)

Antifungal treatment was administered in 85% (51 out of 60 patients) of PA patients. PA patients were divided into 3 subgroups according to antifungal treatment time: 9 patients without antifungal therapy were considered subgroup 1, 10 patients who received antifungal therapy before *Aspergillus* isolation were considered subgroup 2, and 41 patients who received antifungal therapy after positive *Aspergillus* isolation were considered subgroup 3. The most commonly used antifungal drug was voriconazole (46, 76.7%), followed by caspofungin (24, 40%), amphotericin B (4, 6.7%), and itraconazole (3, 5%). Among the three subgroups, the overall in-hospital mortality was 77.8%, 30% and 41.5%, respectively (*p* = 0.094). There was a significant difference in 180-day mortality among the three subgroups (88.9% vs 30% vs 50%, *p* = 0.034). During the follow-up period of 180 days, group 2 had a significantly higher cumulative survival rate than groups 1 and 3 (Fig. 3, *p* = 0.001). Fifty-one PA patients treated with antifungals received monotherapy (n = 29) or combination therapy (n = 22). Twelve patients in the monotherapy group and 11 patients in the combination therapy group died during the follow-up period. Figure 4 showed no difference observed in the 180-day prognosis between the monotherapy and combination therapy groups (log-rank, *p* = 0.427).

## Discussion

This real-world study investigated the clinical significance of *Aspergillus* spp. isolation from LRT specimens in a cohort of AECOPD patients considering three aspects: (1) the incidence of infection and colonization in patients with *Aspergillus* isolation from LRT specimens; (2) differences between infection and colonization in terms of clinical characteristics; and (3) the prognostic factors of the *Aspergillus* infection and the effects of antifungals on prognosis.

It is important for physicians to distinguish infection from colonization in AECOPD patients when *Aspergillus* is isolated from LRT specimens since an early diagnosis is crucial to improve the prognosis [14]. Some studies have demonstrated that once admitted to the hospital, 1.3%-3.9% of patients with COPD develop culture-positive invasive aspergillosis, not always as a result of oral corticosteroid use [16]. Our study revealed that the LRT specimens from 6.63% of AECOPD patients were positive for *Aspergillus*, and most of them had *Aspergillus* infections, resulting in a PA incidence rate of 5.3% in hospitalized AECOPD patients. Among studies focused on COPD patients with *Aspergillus* isolated from LRT specimens, the actual prevalence of infection varied from 22.2 to 61.2% [7, 17, 18]. In our study, *Aspergillus* isolation from LRT specimens from AECOPD patients revealed that 80% of patients were infected with *Aspergillus*. Thus, *Aspergillus* isolation from LRT specimens in hospitalized AECOPD is important and meaningful. In most cases, isolation indicates *Aspergillus* infection rather than colonization.

Uncovering risk factors for infection is important in differentiating between infection and colonization [19]. In terms of clinical symptoms, only fever was significantly more common in the PA group than in the colonization group. Cough and sputum production were also observed in most AECOPD patients in this study. However, cough and sputum were not graded in this study. As a result, the actual differences may be underestimated.

Our study found that systemic corticosteroid use was more common in PA patients than in colonization patients. Many other studies have shown that the utility of corticosteroids plays a significant role in *Aspergillus* colonization [20] and the emergence of invasive aspergillosis [14] in patients with COPD, a high dose of corticosteroids is considered a risk factor for IPA [21, 22]. We reported that systemic steroids more than 700 mg was not only possible risk factor for *Aspergillus* infection, but also associated with higher day-180 mortality.

This study illustrated that AECOPD patients with *Aspergillus* infection had a higher rate of broad-spectrum antibiotic use prior to admission than patients with colonization. This result was also found in other studies [12, 22]. Antibiotic use may play a role in predisposing patients to *Aspergillus* colonization and infection. When antibiotic treatment fails in AECOPD patients, physicians should consider the probability of *Aspergillus* infection. Although the proportion of aspergillosis patients with GOLD III-IV lung function was higher than that of colonization patients, the relationship between lung function and *Aspergillus* infection remains unclear.

The efficiency of BALF and serum GM detection for the early diagnosis of *Aspergillus* infection has been clearly demonstrated in neutropenia patients [23]. A meta-analysis suggested that serum GM had a lower positivity rate in immunocompetent hosts (61–71%) [24]. The data in our study seem to confirm that serum GM detection is not satisfactory. In nonneutropenic patients, BALF GM is more sensitive than serum GM [25, 26]. Our previous study indicated that when the BALF GM cutoff value was set to ≥ 0.5 or ≥ 1.0, the positive-likelihood ratios of the BALF GM test were all higher than those of the serum GM test [27].

The timely initiation of antifungal treatment is important in invasive fungal disease (IFD) [23]. The beneficial effect was expected from antifungal therapy for *Aspergillus* infection in our cohort. Voriconazole is a first-line antifungal for pulmonary aspergillosis [28]. In our study, 76.7% of PA patients were treated with voriconazole. Although the prognosis of patients with monotherapy and combination therapy was not different in our study, combination antifungals are suggested for cases of severe invasive fungal infections [29].

It is well-known that IPA is a threatening disease, while few studies focus on significance of *Aspergillus* positive culture for diagnosing *Aspergillus* infection. Our study reminds clinicians of high incidence of AECOPD patients. Our results also put forward the need of antifungal treatment initiation for at-risk patients as soon as they receive steroids more than 700 mg or present sign of respiratory failure at admission.

This real-world study has some limitations. First, not all COPD patients received underwent LRT specimen examinations, which limited the number of samples. Second, histopathological examination is necessary to confirm the diagnosis. However, AECOPD patients were not able to undergo invasive procedures due to poor lung function.

In summary, it is of great significance to isolate *Aspergillus* from LRT specimens from AECOPD patients. AECOPD patients with PA have a much higher risk of mortality than those with *Aspergillus* colonization. Antifungal treatment is associated with improved survival whereas systemic steroids and respiratory failure are associated with poor prognosis. Early diagnosis of PA by sensitive microbiological tests and an effective algorithm is pivotal to improve survival.

## Data Availability

The datasets used and/or analysed during the current study are available from the corresponding author on reasonable request.

## Abbreviations

LRT: Lower respiratory tract
AECOPD: Acute exacerbation of chronic obstructive pulmonary disease
PA: Pulmonary aspergillosis
HR: Hazard ratio
ABPA: Allergic bronchopulmonary aspergillosis
IPA: Invasive pulmonary aspergillosis
CPA: Chronic pulmonary aspergillosis
COPD: Chronic obstructive pulmonary disease
ECSMID: European Society for Clinical Microbiology and Infectious Diseases
ERS: European Respiratory Society
GM: Galactomannan
CT: Computed tomography
GOLD: Global initiative for chronic obstructive lung disease
ICU: Intensive care unit
WBC: White blood cell
CRP: C-reactive protein
PCT: Procalcitonin
ALB: Albumin
BALF: Bronchoalveolar lavage fluid
CI: Confidence interval
